# Hyperbaric oxygen preconditioning and the role of NADPH oxidase inhibition in postischemic acute kidney injury induced in spontaneously hypertensive rats

**DOI:** 10.1371/journal.pone.0226974

**Published:** 2020-01-08

**Authors:** Sanjin Kovacevic, Milan Ivanov, Zoran Miloradovic, Predrag Brkic, Una Jovana Vajic, Maja Zivotic, Nevena Mihailovic-Stanojevic, Djurdjica Jovovic, Danijela Karanovic, Rada Jeremic, Jelena Nesovic-Ostojic

**Affiliations:** 1 Department of Pathophysiology, Medical Faculty, University of Belgrade, Belgrade, Serbia; 2 Institute for Medical Research, Department of Cardiovascular Physiology, University of Belgrade, Belgrade, Serbia; 3 Department of Medical Physiology, Medical Faculty, University of Belgrade, Belgrade, Serbia; 4 Department of Pathology, Medical Faculty, University of Belgrade, Belgrade, Serbia; Max Delbruck Centrum fur Molekulare Medizin Berlin Buch, GERMANY

## Abstract

Renal ischemia/reperfusion injury is a common cause of acute kidney injury (AKI) and hypertension might contribute to the increased incidence of AKI. The purpose of this study was to investigate the effects of single and combined hyperbaric oxygen (HBO) preconditioning and NADPH oxidase inhibition on oxidative stress, kidney function and structure in spontaneously hypertensive rats (SHR) after renal ischemia reperfusion injury. HBO preconditioning was performed by exposing to pure oxygen (2.026 bar) twice a day for two consecutive days for 60 minutes, and 24h before AKI induction. For AKI induction, the right kidney was removed and ischemia was performed by clamping the left renal artery for 45 minutes. NADPH oxidase inhibition was induced by apocynin (40 mg/kg b.m., intravenously) 5 minutes before reperfusion. AKI significantly increased renal vascular resistance and reduced renal blood flow, which were significantly improved after apocynin treatment. Also, HBO preconditioning, with or without apocynin treatment showed improvement on renal hemodynamics. AKI significantly increased plasma creatinine, urea, phosphate levels and lipid peroxidation in plasma. Remarkable improvement, with decrease in creatinine, urea and phosphate levels was observed in all treated groups. HBO preconditioning, solitary or with apocynin treatment decreased lipid peroxidation in plasma caused by AKI induction. Also, combined with apocynin, it increased catalase activity and solitary, glutathione reductase enzyme activity in erythrocytes. While AKI induction significantly increased plasma KIM– 1 levels, HBO preconditioning, solitary or with apocynin decreased its levels. Considering renal morphology, significant morphological alterations present after AKI induction were significantly improved in all treated groups with reduced tubular dilatation, tubular necrosis in the cortico-medullary zone and PAS positive cast formation. Our results reveal that NADPH oxidase inhibition and hyperbaric oxygen preconditioning, with or without NADPH oxidase inhibition may have beneficial effects, but their protective role should be evaluated in further studies.

## Introduction

Acute kidney injury (AKI) is associated with significant in-hospital morbidity and mortality, particularly in those admitted to the Intensive care units, where mortality rates may exceed 50% [1]. Besides increased mortality rates, there are chronic consequences that carry high risk of developing or exacerbating chronic kidney disease and accelerated development of the end-stage renal disease [2]. Several factors, such as hypertension [3], invasive diagnostic procedures and complex surgery, especially cardiothoracic surgery, partial nephrectomy, renal transplantation or renal stone surgery might contribute to the increased incidence of AKI [4].

Renal ischemia/reperfusion injury is a common cause of AKI [5–7]. The pathophysiology of ischemic AKI is multifactorial and complex and includes increased oxidative stress, hemodynamic alterations, inflammation, endothelial and epithelial injury, followed by reperfusion injury [1]. A common link between AKI and hypertension is enhanced generation of reactive oxygen species (ROS) during injury/disease progression, where NADPH oxidase, one of the major ROS generators, has important role in both conditions [5]. Oxidative stress has a very important role in renal damage, and opens potential targets for therapeutic intervention. It both directly and indirectly affects all aspects of the kidney, including vascular reactivity, renal hemodynamics, glomerular filtration, tubular reabsorption and secretion in all nephron segments [8].

Apocynin (APO, 4-hydroxy-3-methoxyacetophenone) is an efficient inhibitor of NADPH oxidase. It shows antioxidant and anti-inflammatory effects and has been used in many experimental models [9, 10]. Actually, precise mechanism of NADPH inhibition is still incompletely defined, but involves the impairment of the intracellular translocation of two critical cytosolic components of the NADPH-oxidase complex present in cell membrane. It also involves activation by myeloperoxidase, because the agents that promote release of this enzyme enhance efficacy of apocynin, while inhibition is absent in cells devoid or deficient of myeloperoxidase[10].

Hyperbaric oxygenation (HBO) has been used as a primary or adjunctive therapy over the last 50 years. HBO is used to increase blood oxygen level that can penetrate to ischemic areas and perilesioned tissue more deeply than under normobaric conditions and has found its place, as primary or adjuvant therapy in the treatment protocols for different clinical conditions [11–13]. Experimental studies and clinical observations support the evidence that HBO preconditioning has beneficial effect in postischemic reperfusion injury [12–14]. It improves oxygen delivery to an area with diminished circulation, resulting in improved endothelial function and decreased local inflammation and edema. Also, it may directly affect gene expression, signal transduction and cell apoptosis [14]. It affects an antioxidant enzyme expression and the time between two exposures can be observed as pseudohypoxia which is important for upregulation of antioxidative enzymes. In traumatic brain injury, HBO attenuates inflammatory responses by limiting production of inflammatory mediators, creating better environment for repair and regeneration [12]. However, these effects are dependent on treatment parameters–pressure and duration on the treatment [15, 16].

Up to date no effective treatment for AKI is available. Considering the complexity of of AKI pathogenesis and the uprising prevalence of hypertension worldwide, it is reasonable to assume that only combination of different treatment protocols could provide better prognosis for recovery of postischemic AKI [17].

Taking all these things into consideration, the aim of this study was to evaluate the effects of single and combined HBO preconditioning and NADPH oxidase inhibition on oxidative stress, kidney function and structure in spontaneously hypertensive rats with postischemic AKI.

## Material and methods

### Ethics statement

The experimental protocol was approved by the Ethic Committee of the Institute for Medical Research, University of Belgrade, Serbia (No. (No. 323-0702569/2018-05/2), according to the National Law on Animal Welfare ("Službeni Glasnik" no. 41/09) that is consistent with guidelines for animal research and principles of the European Convention for the Protection of Vertebrate Animals Used for Experimental and Other Purposes (Official Daily N. L 358/1-358/6, 18, December 1986) and Directive on the protection of animals used for scientific purposes (Directive 2010/63/EU of the European Parliament and of the Council, 22.9.2010.).

### Animals

Male spontaneously hypertensive rats (SHR, descendants of breeders originally obtained through Taconic Farms, Germantown, NY, USA) 24 weeks old and about 300 g weight were used in this study. The animals were bred at the Institute for Medical Research, University of Belgrade, Serbia, and kept under controlled laboratory conditions (constant temperature 22 ± 1°C, humidity of 65± 1%, 12 h light/dark cycle). The animals were housed in groups of four rats per cage and fed with a standard chow for laboratory rats (Veterinarski zavod, Subotica, Serbia) with free access to food and water. All experimental animals were monitored at least once per day, including weekends and public holidays, throughout the course of the study.

### Experimental design

Hypertension was confirmed in all rats by indirect measurement on tail artery (Narco Bio Systems INC, Houston, TX, USA). The animals were randomly divided into five experimental groups: sham-operated rats (SHAM, n = 9), rats with induced postischemic AKI (AKI, n = 11), animals with AKI and apocynin treatment (AKI+APO, n = 11), group with HBO preconditioning before AKI inducing (AKI+HBO, n = 14) and group with HBO preconditioning before and apocynin treatment after AKI induction (AKI+APO+HBO, n = 13).

Animals with HBO preconditioning were placed into custom made experimental HBO chamber (Holywell Neopren, Belgrade, Serbia) and exposed to 100% oxygen according to the following protocol: 10 minutes of slow compression, 2.0 atmospheres absolute (ATA) for 60 minutes, and 10 minutes of slow decompression, twice a day, at 12 hours interval, during two day period and 24 hours before AKI induction. Upon reaching the desired pressure, the flow of oxygen was reduced to maintain constant pressure while allowing the flow out of the chamber. This constant exchange accompanied by a tray of calcium carbonate crystals was used to reduce the accumulation of CO_2_ in the chamber environment. This protocol corresponds to a standard hyperbaric oxygen treatment that is routinely used in the clinical setting of Center for Hyperbaric Medicine, Belgrade, Serbia [18], and is in a line with recommendations of The Committee of the Undersea and Hyperbaric Medical Society [19]. Each exposure was started at the same hour to exclude any confounding issues associated with the changes in biological rhythm. Body temperature was not changed significantly after the HBOT. Rats were proceeding to AKI induction 12 hours after the last HBO preconditioning.

All surgical procedures were performed in anaesthetized rats by injecting 35 mg/kg b.m. sodium pentobarbital intraperitoneally. AKI was induced by removal of the right kidney and atraumatic clamp occlusion of the left renal artery for 45 minutes. In sham-operated group identical surgical procedure was performed, but without left renal artery clamping. NADPH oxidase inhibitor, Apocynin (Sigma Aldrich, USA), was applied as a bolus injection 5 minutes before clamp removal, 40 mg/kg b.m., intravenously. Animals without apocynin treatment received bolus of vehicle (saline, 50 μl, intravenously). At the end of AKI procedure, the wound abdominal incision was satured and rats were placed into metabolic cages for 24 hours, with free access to food and water. In order to alleviate postoperative pain, ketoprofen (5 mg/kg b.m.) was administrated subcutaneously.

### Hemodynamic measurements

All hemodynamic parameters were measured 24 hours after reperfusion. Measurement of systemic hemodynamic parameters was conducted by a direct method, through a femoral artery catheter (PE-50, Clay-Adams Parsippany, NY, USA), connected to a physiological data acquisition system (9800TCR Cardiomax III-TCR, Columbus, OH, USA). A jugular vein was cannulated with PE-50 for the injection of cold saline. For the determination of cardiac output (CO), the left carotid artery was catheterized with PE-50 tubing and attached to a thermo sensor, which was coupled to the Cardiomax III. The other end of the thermocouple was placed in cold saline. Following 20 min for stabilization after surgery, cold saline (0.2 ml) was supplied through the jugular vein and mean arterial pressure (MAP), heart rate (HR), and CO were recorded. Total peripheral vascular resistance (TPVR) was calculated from MAP and CO (assuming that mean right arterial pressure is zero) and expressed as mmHg x min x kg/ml.

After abdominal incision and left renal artery preparation, renal blood flow (RBF) was recorded by using T106 Small Animal Flowmeter (Transonic System Inc., Ithaca, NY, USA). Renal vascular resistance (RVR) was calculated by dividing MAP with total blood flow through the renal artery, normalized for the body weight and expressed as mmHg x min x kg/ml.

### Sample collection

After hemodynamic measurements, blood samples, obtained by puncture of the abdominal aorta were collected into tubes containing lithium-heparin (Li-heparin, Sigma-Aldrich, USA) and used for further analysis. Blood was centrifuged to separate plasma from erythrocytes. Until assaying, plasma samples were stored at -20°C and erythrocytes samples at -80°C. After blood samples collection, animals were sacrificed by pentobarbital overdose injection. For determination of morphological changes, kidney tissue was removed immediately after sacrificing and then prepared for histological examination.

### Biochemical analyses

Kidney function parameters, including plasma creatinine (PCr), urea (PU), phosphate (PPhos), were measured using the automatic COBAS INTEGRA 400 plus (Hoffmann-La Roche, Germany) analyser.

### Measurement of oxidative stress parameters

#### Determination of lipid peroxidation

In order to determine the degree of lipid peroxidation, the concentration of thiobarbituric acid reactive substances (TBARS) was measured in plasma. TBARS assay was done by using 2-thiobarbituric acid (2,6-dihydrooxypyrimidine-2-thiol; TBA, Acros, Organic), as previously described [20].

#### Determination of erythrocytes antioxidant enzyme activities

Antioxidant enzyme activities of the erythrocytes, catalase (CAT), glutathione reductase (GR), superoxide dismutase (SOD) and glutathione peroxidase (GSH-Px) were measured by spectrophotometry, according to previously described methods [20].

### Determination of plasma kidney injury molecule—1 levels

Plasma kidney injury molecule– 1 (KIM-1) was determined by sandwich enzyme-linked immunosorbent assay (ELISA) (R&D Systems, Inc). The detection range for KIM-1 was 7.8–500 pg/ml.

### Histological examination

For light microscopy observation, the renal tissue was fixed in 10% buffered formalin solution. Later, the kidney was dehydrated in alcohol and embedded in paraffin block, cut into 5μm thick sections and stained by hematoxiline eosine (HE) and periodic acid-Schiff (PAS) reagent. Slides were examined by two independent pathologists blinded to the experimental protocol. By light microscopy, according to the degree of lesions, following parameters were semi quantitatively evaluated: morphological alterations in renal tubular cells, on the scale from 0 to 4 (0 –normal tubular cells, 1 –loss of luminal membrane or brush borders, 2 –swelling and vacuolization of cells, 3 –separation of the cells from tubular basal membrane, 4 –as previous with complete loss of epithelial tubular cells); tubular dilatation and presence of intraluminal PAS positive cast formations were graded same on scale from 0 to 3 (0 –without tubular dilatation/PAS positive cast formations, 1 –up to 10%, 2 –up to 30%, 3 –more than 30% tubules dilated/PAS positive cast present). The sum of these changes represented the histopathological score for each group which was used for comparison between groups.

### Statistical analysis

All data are expressed as the mean ± standard deviation (SD). A statistical analysis of each of the parameters of interest was carried out using analysis Student’s t-test for independent samples and analysis of variance (One–way ANOVA). Animals in AKI group were compared to SHAM operated rats using Student’s t-test for independent samples. P value <0.05 was considered significant. Animals in APO, HBO and APO+HBO group were compared to AKI using One–way ANOVA. When a significant F value in One–way ANOVA test (p<0.05) was obtained, post hoc test (Dunnett’s multiple comparisons test) was used. The statistical calculations were performed using GraphPad Prism for Windows (Version 7.0, GraphPad Software, La Jolla California, USA).

## Results

### Hemodynamic parameters

Systemic hemodynamics values, 24 hours after reperfusion, are shown in Table 1. MAP was significantly decreased in AKI group compared to SHAM group (p<0.001). Also, in AKI group HR was significantly decreased in comparison to SHAM (p<0.05), without differences in CO and TVPR. APO treatment significantly decreased TVPR (p<0.05), but without significant impact on MAP, HR and CO vs. AKI. In groups with HBO preconditioning, with or without APO treatment TVPR was significantly decreased compared to control AKI group, too (p<0.05).

**Table 1:** Systemic hemodynamic parameters.

	SHAM (n = 9)§	AKI (n = 11)	AKI+APO (n = 11)§	AKI+HBO (n = 14)§	AKI+APO+HBO (n = 7)
MAP (mmHg)	133.11±14.90	102.18±15.28***	91.45±18.46	97.71±20.06	102.29±18.06
HR (beat/min)	435.33±17.77	366.91±92.27*	387.55±27.15	343.43±73.82	397.29±19.13
TVPR (mmHg x min x kg/ml)	0.51±0.24	0.57±0.36	0.33±0.12^#^	0.36±0.08^#^	0.32±0.06^#^
CO (ml/min/kg)	414.75±146.20	394.32±146.00	444.72±121.90	400.09±98.72	470.41±43.51

MAP-mean arterial pressure, HR-hearth rate, TVPR-Total peripheral vascular resistance, CO-cardiac output, n–number of animals.

^§^ for TVPR and CO, n = 8 (SHAM), n = 10 (AKI + APO), n = 12 (AKI+HBO),

*p<0.05,

***p<0.001 vs. SHAM group;

^#^p<0.05 vs. AKI group

**Fig 1:**
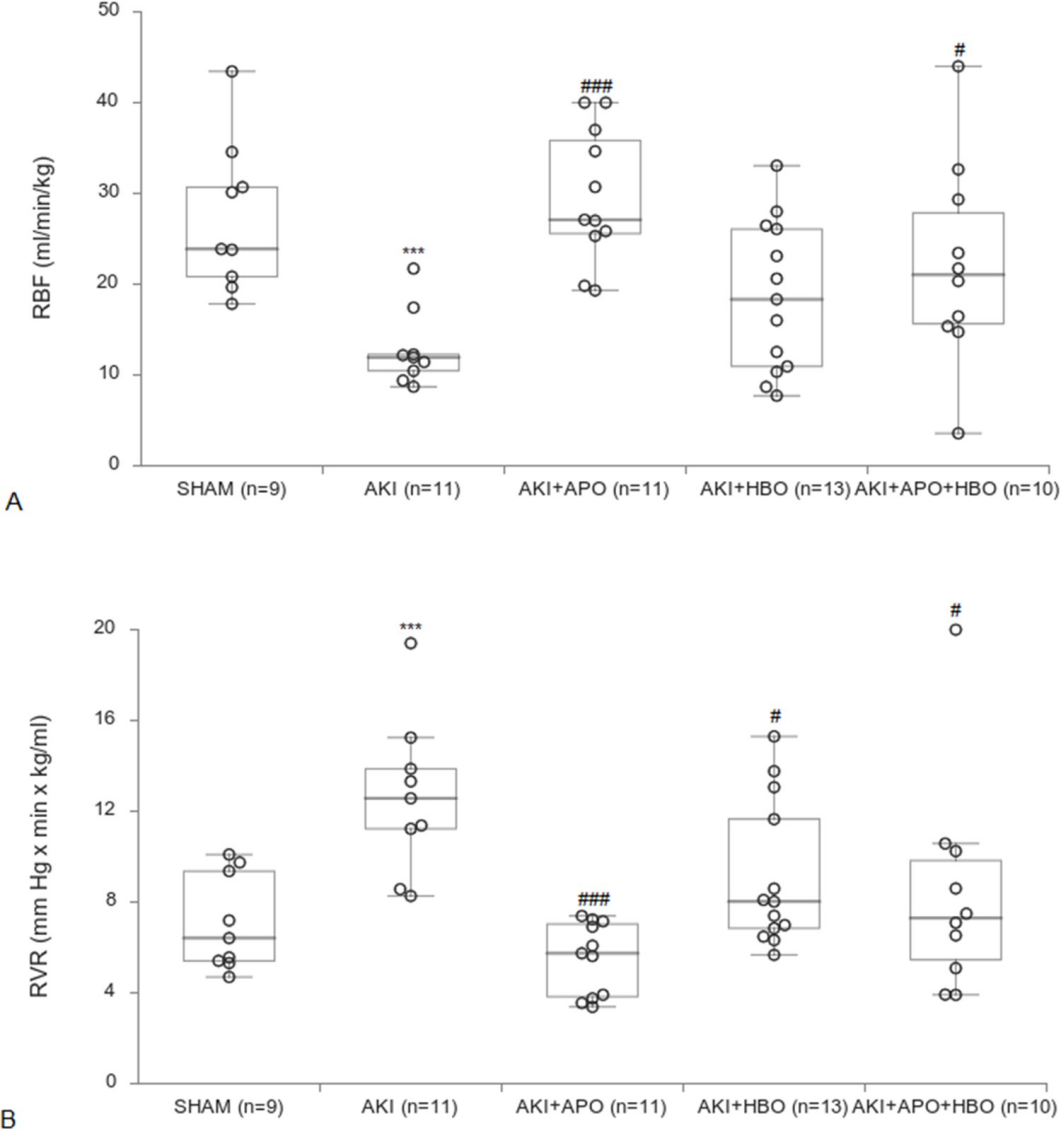
Renal hemodynamic parameters. RVR-renal vascular resistance, RBF-renal blood flow. ***p<0.001 vs. SHAM group, #p<0.05, ###p<0.001 vs. AKI group.

Considering renal hemodinamics, AKI significantly reduced RBF and increased RVR in comparison to SHAM (RBF—p<0.001; RVR—p<0.001), while in APO group significant improvement was found compared to AKI (RBF—p<0.001; RVR—p<0.001). HBO preconditioning with (RBF—p<0.05; RVR—p<0.05) or without APO treatment (; RVR—p<0.05) also showed improvement on renal hemodinamics in comparison to AKI (Fig 1A and 1B).

### Biochemical parameters

**Fig 2:**
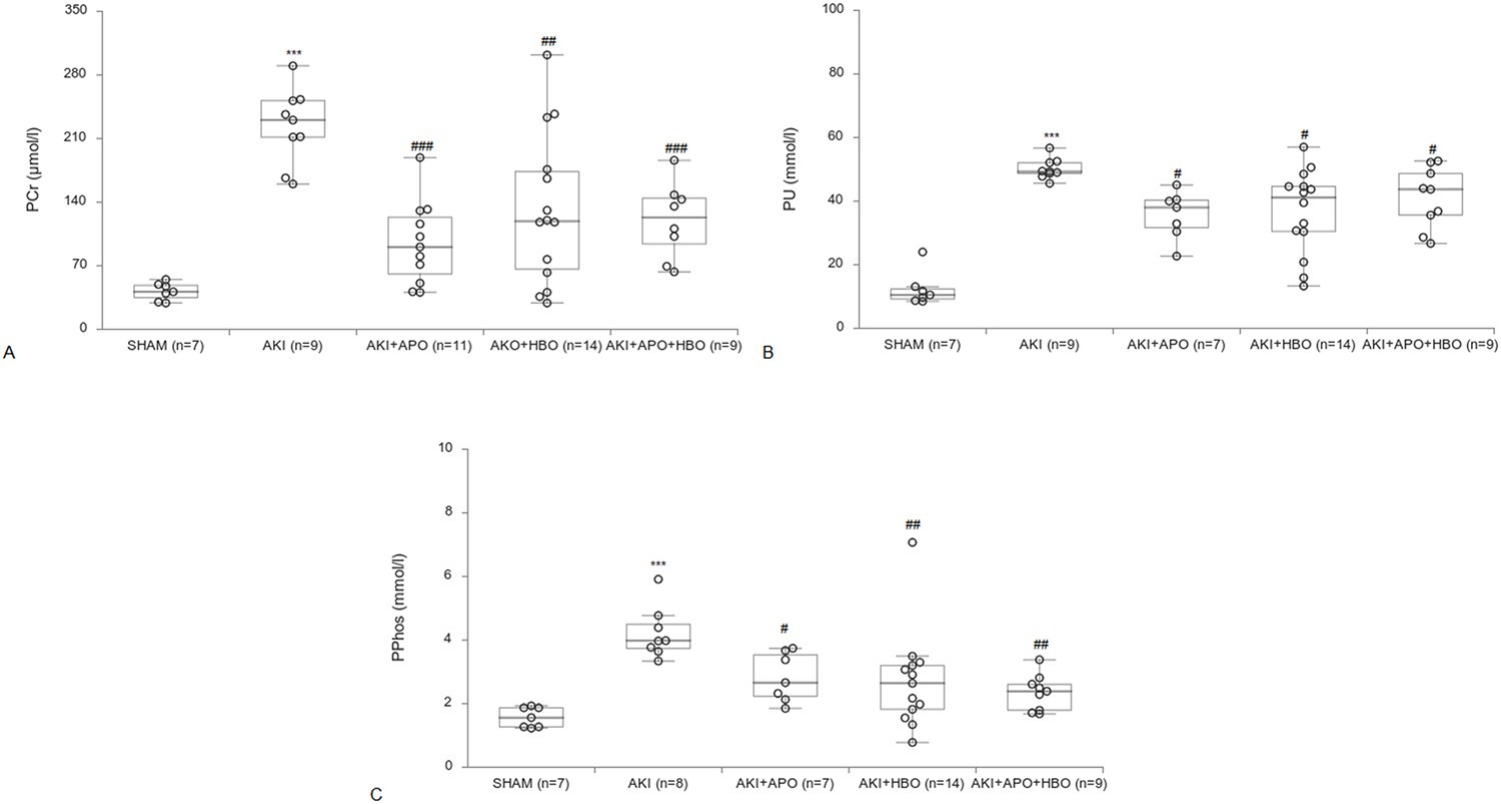
Plasma creatinine (PCr), urea (PU) and phosphate (PPhos) concentration 24 h after reperfusion. ***p<0.001 vs. SHAM group, #p<0.05, ##p<0.01, ###p<0.001 vs. AKI group.

PCr, Pu, PPhos concentration are shown in Fig 2. AKI induction significantly increased total PCr, Pu and PPhos levels when compared to SHAM group. Remarkable decrease in PCr levels were observed in groups with APO treatment (p<0.001), HBO preconditioning and APO treatment (p<0.001) and HBO preconditioning solitary (p<0.01, Fig 2A). Other two kidney function parameters (PU, PPhos) in treated groups also showed significant improvement in comparison to AKI control group (Fig 2B and 2C).

### Oxidative stress parameters

#### Lipid peroxidation

**Fig 3:**
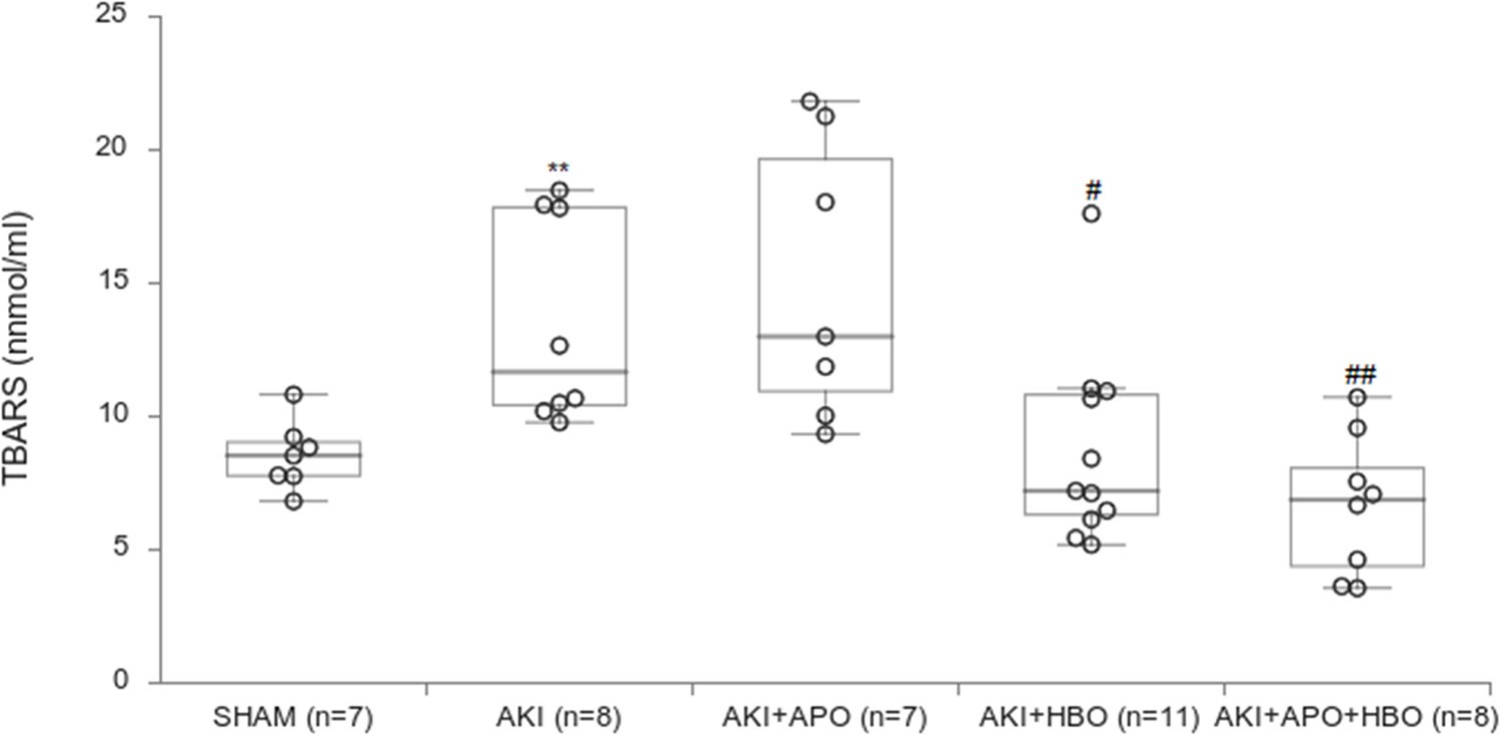
Plasma TBARS levels 24 hours after reperfusion. **p<0.01 vs. SHAM group, #p<0.05, ##p<0.01vs. AKI group.

Level of TBARS, as marker of lipid peroxidation, is shown in Fig 3. In AKI group there was a significant increase (p<0.01) in TBARS level, compared to SHAM group. Preconditioning with HBO therapy significantly decreased lipid peroxidation in plasma in comparison to AKI (AKI+HBO-p<0.05; AKI+HBO+APO-p<0.01). There was no difference in TBARS level between AKI and AKI+APO groups.

#### Antioxidant enzymes

**Fig 4:**
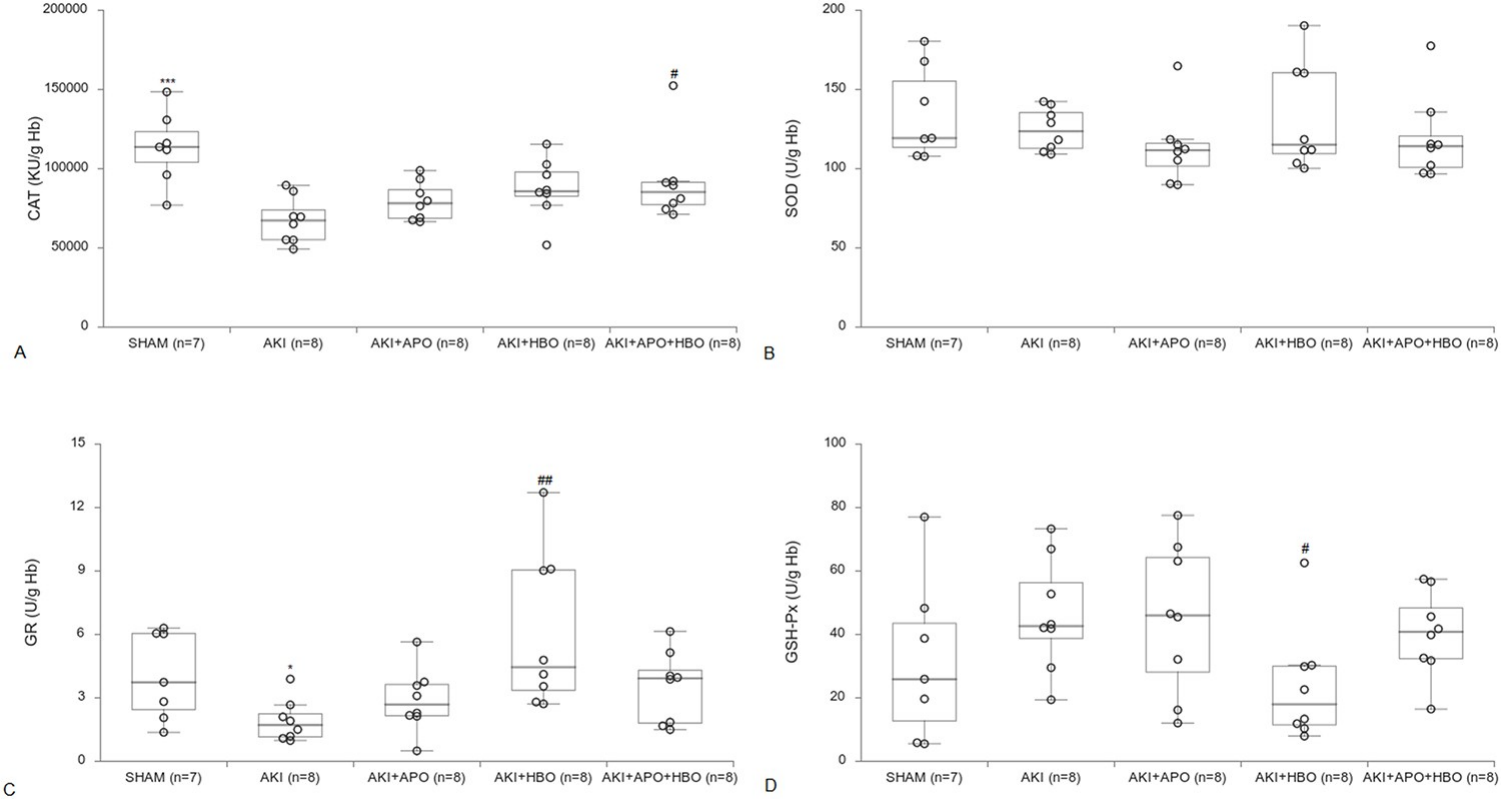
Erythrocytes catalase (CAT), superoxide dismutase (SOD), glutathione peroxidase (GSH-Px) and glutathione reductase (GR) activity 24 hours after reperfusion. *p<0.05,***p<0.001 vs. SHAM, #p<0.05, ##p<0.01 vs. AKI.

CAT activity dropped after AKI induction (p<0.001), while in AKI+APO+HBO group its activity was significantly elevated in comparison to AKI (Fig 4A; p<0.05). In SOD activity there was no significant difference among the groups (Fig 4B). In AKI group GR activity was significantly lower compared to SHAM (p<0.05), while in comparison to AKI, enzyme activity increased in HBO treated group (p<0.01; Fig 4C). GSH-Px activity significantly decreased in group with hyperbaric oxygen preconditioning (p<0.05), with no differences among the other groups (Fig 4D).

### Plasma KIM—1 levels

**Fig 5:**
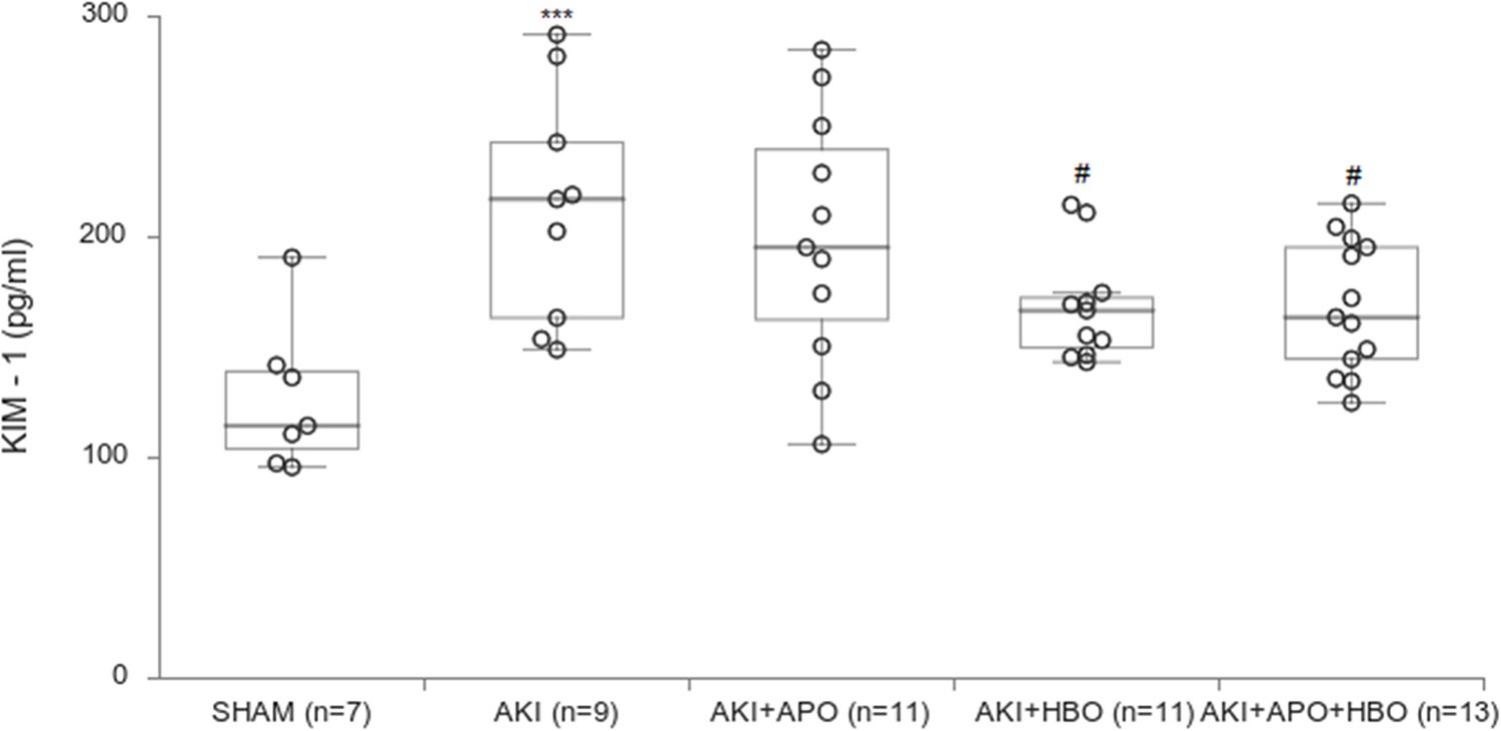
Plasma KIM—1 levels 24h after reperfusion. *** p<0.001 vs. SHAM, # p<0.05 vs. AKI.

KIM-1 levels were significantly increased in AKI group compared to SHAM operated rats (p<0.001). Remarkable decrease in KIM– 1 levels was noticed in groups with HBO preconditioning (p<0.05) and HBO preconditioning with APO treatment (p<0.05), while in APO group this decrease was not significant (p>0.05, Fig 5).

### Histological findings

**Fig 6:**
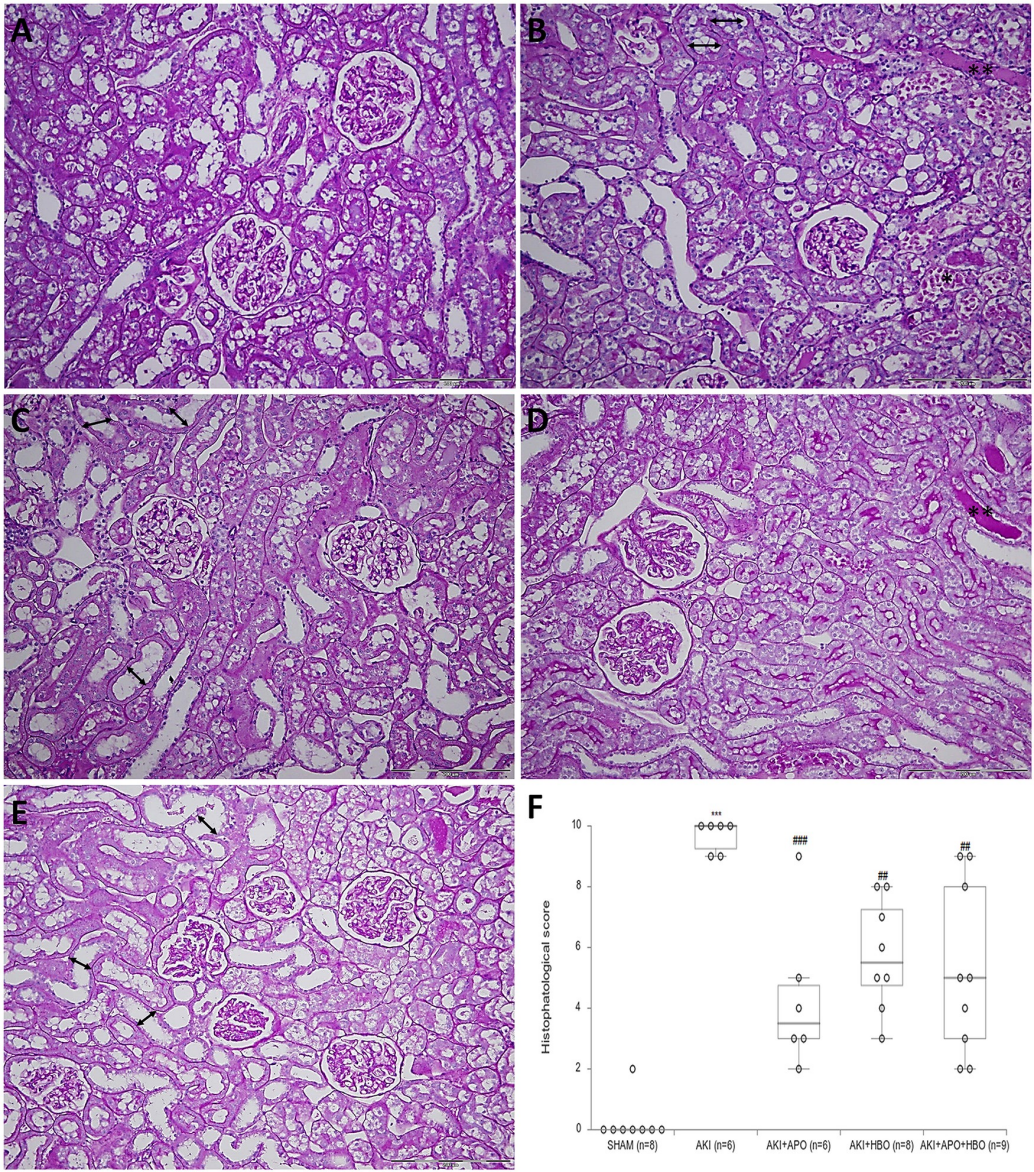
Histopathology of the representative kidney samples collected in different experimental groups (PAS staining, x 20 magnification): Normal morphology of renal tissue including glomerular and tubulointerstitial compartments in the sham-operated animals (A), proximal tubular dilatation (arrows), necrosis of tubular epithelial cells (*) and PAS-positive casts (**) in the AKI group (B), moderately intensive tubular necrosis, reduced tubular dilatation and less number of PAS-positive casts in AKI+APO (C), AKI+HBO (D), AKI+APO+HBO (E) groups and histopathological score as a sum of present morphological changes (F). ***p<0.001 vs. SHAM, ##p<0.01, ###p<0.001 vs. AKI.

By comparison between the groups, significant differences in morphological alterations were noticed. In SHAM operated rats, normal morphology of glomeruli, tubulointerstitium, and blood vessels were observed including rare kidney specimens with a few PAS positive casts in the lumen of the tubules (Fig 6A). In animals with AKI significant morphological alterations were present: tubular cells necrosis, dilatation of certain segments of the proximal and distal tubules, mostly with loss of brush-border. The most notable changes were present in the cortico-medullary zone, where the broad areas of tubular necrosis and a large number of PAS positive casts in the collecting ducts were observed (Fig 6B). In treated animals (AKI+APO, AKI+HBO, AKI+APO+HBO) degrees of morphological changes were significantly lower compared to AKI control. There were reduced tubular dilatation, tubular necrosis in the cortico-medullary zone and PAS positive cast formation (Fig 6C, 6D and 6E). In addition, the histopathological score (Fig 6F), as a sum of these changes was significantly higher in AKI group, compared to SHAM control. In treated groups, this score was significantly lower in comparison to AKI (AKI vs. AKI+APO, p<0.001; AKI vs. AKI+HBO, p<0.01; AKI vs. AKI+APO+HBO, p<0.01).

## Discussion

In this study has shown for the first time, that HBO preconditioning improves oxidative defense in SHR, as well as, renal function and structure of hypertensive kidneys suffered postischemic AKI. Further, we showed that NADPH oxidase has important role in development and maintenance of AKI in hypertensive surrounding.

Hypertension is a condition with enhanced ROS generation [21]. Postischemic AKI is also accompanied with increased plasma lipid peroxidation and oxidative stress [6, 7]. We showed that both groups with HBO preconditioning, reduced lipid peroxidation. The therapeutic basis behind HBO preconditioning is a significant increase in oxygen concentrations within different tissues, which can increase production of ROS [22, 23]. Paradoxically, it is also an important stimulus to upregulate antioxidant enzymes in response to a greater production of ROS [22, 23]. In accordance, in present study, HBO preconditioning in combination with apocynin increased plasma catalase activity. Also, gluthatione reductase activity was increased in HBO group. Kim et al. demonstrated that hyperbaric oxygen pretreatment induces catalase in the *in vivo* heart and reduces infarct size in ischemic rat myocardium [24]. In those pathological conditions catalase activity may be crucial for a protective effect against oxidative stress. In Moreno et al. study, it is found that intensely stained CAT-immunoreactive cells (corresponded to neurons) were resistant to ischemia reperfusion injury, whereas, weakly stained cells were more susceptible to ischemic damage [25]. However, APO treatment solitary did not reduce plasma lipid peroxidation nor affect erythrocytes antioxidant enzyme activities in treated animals. Despite APO has traditionally used as NADPH oxidase inhibitor, to be effective it must be converted to an active dimer by myeloperoxidase [26]. Studies on HEK-293 cells showed that APO is not an NADPH oxidase inhibitor in myeloperoxidase free vascular cells [27], which can partially explain lack of lipid peroxidation reduction. In contrary, it was shown that APO could be an effective tool that inactivates NADPH oxidase in renal tissue markedly reducing lipid peroxidation [28]. Activity of other antioxidant enzymes, SOD and GSH-Px were unaffected by AKI or in our treated groups. Nevertheless, in hypertensive patients, disturbances in glutathione related antioxidant defense are reported [29]. It has been shown that glutathione peroxidase can be inactivated in the conditions of oxidative stress where superoxide radical may inhibit enzyme peroxide function and in hypertension pathogenesis superoxide radical plays major role [29]. Also, SOD activity can be inactivated due to enhanced production of oxidants, through modifications of the enzyme active site [30]. These results are in a line with unaffected SOD activity in the present study [5].

ROS production have direct effects on vasoreactivity in renal vessels, with increased ROS associated with enhanced renal RVR [6]. On the other hand NADPH oxidase is the major source of ROS in many tissues, including the kidney, especially in AKI [31]. In the present study, we used APO and confirmed that improved RBF, and decreased RVR, are in part, induced by decreased oxidative stress. APO is experimentally used as an inhibitor of vascular NADPH oxidases [32], but there is evidence proving that APO is not an inhibitor of vascular NADPH oxidases, but is an antioxidant [27]. Indeed, Heumüller at al. showed that the inhibitory action of apocynin for NADPH oxidases is restricted to myeloperoxidase—expressing leukocytes and that the compound does not inhibit NADPH oxidases in myeloperoxidase—free vascular cells [27]. The immune response to kidney damage during AKI is an important contributor to reduced renal function and progression of kidney injury [33]. This response is complex, involving numerous proinflammatory myeloperoxidase-expressing leukocytes which employ diverse effector mechanisms inside the kidney, including increasing ROS [34–36]. Further, Ivanov et al. previously explained that hypertension and postischemic acute kidney injury are accompanied with elevated circulating levels of angiotensin II (Ang II). They had observation that NADPH oxidase activity can be stimulated by Ang II. Furthermore, in this study authors demonstrated that in combine model of hypertension and postischemic AKI, kidney oxidative injuries are strongly mediated by Ang II [6]. Taking all together, we can assume that increased oxidative stress, besides increased level of Ang II itself, is very important factor responsible for decreased RBF and increased RVR. Also, as accumulated renal leukocytes in AKI are important source of ROS [33], it is reasonable to assume that ROS are, in part, responsible for increased level of Ang II. It is also possible that APO, by its inhibitory action on NADPH oxidases of myeloperoxsidase—expressing leukocytes is able to decrease the level of Ang II, which will further decrease RVR and improve RBF. On the other hand, Schüter et al., showed that apocynin-induced vasodilatation is the result of Rho kinase inhibition [37], so maybe there are some additional mechanisms of APO that are also involved in RBF improvement and RVR decrease in AKI after APO treatment.

In the present study, we also demonstrated beneficial effect of HBO precondition on renal hamodynamic. Our results are in accordance with Rubinstein at al. who claims that HBO treatment can improve renal hemodynamic, due to the HBO induced renal vasodilatation [38]. Klemetti et al. in their study indicate that short HBO treatment can be successfully used for improving blood flow of healing tissues in rats [39]. Experimental evidence suggests beneficial effects of HBO when used as a preconditioning stimulus in I/R injury. HBO seems to offer a reservoir of oxygen that may last for a few hours and may have great importance in case of sudden hypoxia or ischemia. Besides being carried through by blood, oxygen reaches to the cellular level also by diffusion from the interstitial tissue in which it reaches high concentration during HBO treatment. It improves endothelial function and decreases local inflammation and edema [14]. Also, as increased ROS is associated with enhanced RVR, we can assume that upregulated antioxidant enzyme activities [40,41] after HBO preconditioning may decreased RVR by ROS scavenging that would further improve renal hemodynamic.

Considering obtained results of systemic hemodynamic after AKI induction, decreased MAP and HR were noticed. These results are consistent with Bowmer study [42]. Namely, these authors have brought together a high uremia influence on diminishing α_1_ adrenoreceptors sensitivity with the cause of both MAP and HR reduction after AKI [42]. This correlates with high value of plasma urea in our study, as well. In the presented study in all treated groups TVPR was decreased, without changes in MAP and HR, after AKI induction. Rubinstein at al. showed that in their model of ischemia/reperfusion renal injury, HBO did not affect MAP [39]. However, TVPR decrease in HBO treated groups might be explained by decreased ROS generation. Under normotensive conditions, blood pressure is closely regulated by several endogenous vasodilators and constrictors and does not depend on vascular superoxide to a significant extent. However, in hypertension, shear stress and several hormones induce stimulus dependent ROS production in various vascular cell types, thereby inducing endothelial dysfunction and hypertension [43]. On the other hand, treatment with apocynin can reverse vascular functional and structural changes in experimental hypertension and prevent or reduce blood pressure elevation. Additional mechanism of action associated with apocynin involves increased NO expression and activity [44].

The results of present study showed that ischemia reperfusion led to a significant increase in creatinine, urea and phosphate levels with marked improvement in all treated groups. During renal damage in ischemia reperfusion injury, as a result of diminished glomerular filtration rate, ability of the kidney to filter creatinine and nonprotein waste products is reduced [6]. Moreover, the levels of urea and uric acid are elevated, and hypophosphatemia frequently occurs due to diminished expression of tubular sodium dependent phosphate cotransporter [45]. Different studies indicate that APO could contribute in many ways to improve renal function. By causing the inhibition of NADPH oxidase, APO expressed the ameliorative effect to renal function which may be attributed to the protection against oxidative stress injury. Besides this, APO prevents adenosine triphosphate depletion during ischemia that leads to apoptosis and necrosis of tubular cells [46]. Also, by reducing leukocyte infiltration and production of inflammatory cytokines, such as TNF-α, Il-1β and Il-6, it decreases an acute inflammatory response [47]. These results come in line with study of Altintas et al. who showed improvement in kidney function after ischemia reperfusion injury in normotensive Wistar albino rats [48]. Significant improvement can be seen in other experimental models, followed by renal dysfunction, such as gentamicin-induced nephropathy [49]. HBO preconditioning also improved renal function. Different studies suggest that HBO also has anti-inflammatory effect by decreasing neutrophil infiltration and further tissue damage [50, 51]. Xiaoyhou He et al. showed that HBO preconditioning protocol as we used in this study had beneficial effects on renal function [52].

Since its discovery, KIM-1 has emerged as a sensitive and specific biomarker of kidney injury in both rodent models and humans. KIM-1 may be released into the circulation after kidney proximal tubule injury. First, it is released into the interstitium after tubular cell polarity is lost. Further, increased transepithelial permeability leads to backleak of tubular contents into the circulation with altered microvascular permeability as an important contributing factor [53]. Decreased KIM– 1 plasma levels in AKI+HBO and AKI+APO+HBO groups may indicate minor degree of kidney tissue alterations which is in accordance with our previously shown results and further histophatological findings.

Histopathological examination is the best way to analyze morphological changes in the kidney tissue after AKI episode. Prominent morphological features of ischemic AKI include effacement and loss of proximal tubule brush border, patchy loss of tubule cells, focal areas of proximal tubular dilatation and distal tubule casts, and areas of necrosis are the most notable changes in the ischemic kidney that can be seen by light microscopy [6]. In our study, morphological changes in the kidneys isolated from rats with induced ischemic AKI, exactly correspond to the previous description.

Our results clearly indicate that APO, used to block NADPH oxidase in the early stages of ischemic AKI in hypertension, has beneficial effects on renal morphological structure. Histopathological examination confirmed less severe lesions of tubular epithelial cells, reduced tubular dilatation and less number of PAS-positive casts in treated animals. Altintas et al. [48] showed a protective effect of APO on kidney morphology in Wistar Albino female rats with ischemic AKI.

Numerous studies showed successful recovery potential of HBO therapy in kidney ischemia/reperfusion injuries [38, 52]. In this study, with or without apocynin, treatment with HBO showed beneficial effects on renal morphological structure. Preconditioning with HBO therapy decreased the number of PAS positive casts and the smaller fields of necrosis after ischemic AKI. Rubinstein et al. showed that HBO therapy could have beneficial effects on a kidney structure of male Sprague-Dawley rats 48h after ischemia [38]. Furthermore, He et al. in similar model on male Sprague-Dawley rats suggest that HBO preconditioning has a protective effect on ischemia/reperfusion by reducing the free oxygen radical peroxidation of lipid membranes, and increasing the activity of antioxidative enzymes [52].

These histological findings suggest that positive effect of treatment with HBO and apocynin on morphological level are consistent with the improvement of both, renal artery hemodynamic parameters, as well with better biochemical parameters of kidney function. Furthermore, oxidative stress parameters are in accordance with obtained histological findings suggesting that improved antioxidant defense contributes to morphological protection.

## Conclusion

In conclusion, we showed, for the first time that in experimental conditions, HBO preconditioning as well as NADPH oxidase inhibition with or without HBO preconditioning, improve kidney structure and function, increase renal blood flow, decrease renal vascular resistance and increase antioxidative defense in SHR which suffer AKI. Also, these results suggest that it is reasonable to assume that HBO preconditioning and NADPH oxidase inhibition potentially may have beneficial effects, but further comprehensive experimental and clinical studies are needed to confirm these promising results.

## Supporting information

S1 Table Full data set for systemic hemodynamic parameters in sham-operated rats (SHAM), rats with induced postischemic AKI (AKI), animals with AKI and apocynin treatment (AKI+APO), group with HBO preconditioning before AKI inducing (AKI+HBO) and group with HBO preconditioning before and apocynin treatment after AKI induction (AKI+APO+HBO). MAP (mmHg)-mean arterial pressure, HR (beat/min)-hearth rate, TVPR (mmHg x min x kg/ml)-Total peripheral vascular resistance, CO (ml/min/kg)-cardiac output.
(PDF)
