# Supplementary material for: Hyperbaric oxygen preconditioning and the role of NADPH oxidase inhibition in postischemic acute kidney injury induced in spontaneously hypertensive rats
Source: PLoS One. 2020 Jan 8;15(1):e0226974. doi: 10.1371/journal.pone.0226974 (PMC6948727; doi:10.1371/journal.pone.0226974)
Supplement: S1 Table — MAP (mmHg)-mean arterial pressure, HR (beat/min)-hearth rate, TVPR (mmHg x min x kg/ml)-Total peripheral vascular resistance, CO (ml/min/kg)-cardiac output. (PDF) [file pone.0226974.s001.pdf]

SHAM	AKI	AKI+APO	AKI+HBO	AKI+APO+HBO
150	114	74	106	117
135	91	109	110	110
153	82	74	97	89
130	119	83	99	73
138	95	82	63	125
125	119	98	131	93
143	94	95	77	109
113	119	90	106	
111	114	65	76	
	79	109	97	
	98	127	123	
			105	
			110	
			68	

SHAM	AKI	AKI+APO	AKI+HBO	AKI+APO+HBO
430	400	376	388	413
427	441	427	366	400
424	97	346	200	411
400	356	376	356	366
441	376	427	278	400
457	366	400	175	415
443	397	356	400	376
440	400	366	400	
457	403	388	356	
	402	413	388	
	398	388	366	
			376	
			346	
			413	

SHAM	AKI	AKI+APO	AKI+HBO	AKI+APO+HBO
0.41	0.66	0.33	0.32	0.27
0.28	0.37	0.25	0.44	0.24
0.75	0.62	0.21	0.31	0.36
0.94	0.42	0.41	0.26	0.29
0.33	0.34	0.4	0.41	0.4
0.5	0.27	0.34	0.35	0.34
0.57	0.29	0.24	0.34	0.36
0.28	0.27	0.17	0.29	
	0.73	0.33	0.43	
	1.47	0.58	0.54	
	0.85		0.28	
			0.31	

SHAM	AKI	AKI+APO	AKI+HBO	AKI+APO+HBO
499.16	249.84	332.19	433.33	483.00
647.10	339.13	457.93	334.38	525.29
260.94	143.00	555.69	406.25	492.84
204.09	428.21	395.57	563.33	470.10
400.74	409.68	441.87	401.10	412.65
385.67	568.97	431.62	422.58	410.67
372.55	512.50	400.83	476.67	498.30
547.72	586.67	741.04	340.00	
	209.38	421.87	343.33	
	488.87	266.13	226.67	
	401.30		550.00	
			303.45	
